# Visual impairment in high flow and low flow carotid cavernous fistula

**DOI:** 10.1038/s41598-019-49342-3

**Published:** 2019-09-06

**Authors:** Md. Shahid Alam, Mukesh Jain, Bipasha Mukherjee, Tarun Sharma, Swatee Halbe, Durgasri Jaisankar, Rajiv Raman

**Affiliations:** 1 Department of Orbit Oculoplasty Reconstructive and Aesthetic Services, Sankara Nethralaya, Chennai, India; 2 Shri Bhagwan Mahavir Vitreoretinal Services, Sankara Nethralaya, Chennai, India; 3 Department of Interventional Radiology, Apollo Specialty Hospitals, Vanagram, Chennai India

**Keywords:** Diseases, Eye diseases

## Abstract

Our aim is to study the varied posterior segment manifestations, level of visual impairment (VI) and its causes in carotid cavernous fistula (CCF) patients. A retrospective study was done, wherein data was obtained from 48 digital subtraction angiogram (DSA) proven CCF patients. CCF was classified according to Barrow *et al*., based on DSA into type A (high flow) and types B, C and D (low flow). High flow CCF was present in 8 (16.7%) and low flow CCF was present in 42 (83.3%). Compared to low flow group, patients in high flow group were younger and had a history of trauma (p < 0.05). Posterior segment findings ranged from familiar stasis retinopathy and optic neuropathy (both, glaucomatous and ischemic) to uncommon findings of central retinal artery occlusion, Terson syndrome and combined retinal and choroidal detachment. Retinal vein dilatation was the most common finding in both groups. The high flow CCF group had 6 (75%) patients that had VI. This was acute in 4 (50%) patients and delayed in 2 (25%). In the low flow group 10 (23.8%) of patients had delayed VI. The identification of “3 point sign” is a novel finding of this study, not described before. While none of three findings (disc hyperaemia, retinal vein dilatation and intra-retinal haemorrhage) in isolation were predictive of visual loss, but when present together results in visual loss. Posterior segment changes were varied, some are uncommon and can occur in various combinations. “3 point sign” must be identified at the earliest to prevent visual impairment. The incidence of VI in CCF patients is high.

## Introduction

Carotid cavernous fistulas (CCFs), spontaneous or traumatic, is an abnormal communication between the cavernous sinus and the carotid arterial system^1^. Barrow *et al*. classified CCFs angiographically into four types-type A, B, C and D^2^. In Type A (high flow) CCFs, there is a direct communication between the internal carotid artery-cavernous segment and the cavernous sinus. In Type B, C and D (low flow) CCFs, there is an abnormal communication between the cavernous sinus, and one or more meningeal branches of the internal carotid artery, external carotid artery or both respectively.

CCFs can present with varied presentations: conjunctival chemosis, congestion, proptosis, ptosis, ophthalmoplegia and diplopia, orbital pain, bruit and diminution of vision. Since ocular manifestations are present in the majority of the patients, the ophthalmologists may be the first to encounter these patients^3^. Because of the diverse clinical signs and symptoms with a long list of differentials, CCFs remains undiagnosed in many patients^4^. This can lead to significant morbidity and mortality including visual impairment (VI).

The incidence of VI is high in CCF patients^5,6^. Varied posterior segment changes account to this morbidity. In the literature, although these changes have been described, these are either part of an isolated case report or smaller series where the diagnosis had not been confirmed on DSA^7,8^. Moreover, their incidence and contribution to VI in CCF patients were not analyzed in a large series. We studied the posterior segment manifestations in 48 consecutive cases of CCF, which were proven and classified on Digital subtraction angiography (DSA). We compared the retinal features, levels of VI and its causes among the two groups.

## Study Population and Methodology

### Study design and parameters studied

A retrospective study was done at a tertiary eye care center in South India; wherein the data were obtained from the electronic medical records of all CCF patients seen at our center from June 2001 to June 2015. A total of 48 patients were included in the study. We excluded patients who were suspected to have CCF (on clinical evaluation and initial imaging studies) but did not undergo a DSA and those who received some form of treatment for CCF (carotid massage, embolisation) before their presentation at our clinic.

The Institutional review board of Vision Research Foundation approval was obtained to analyze the hospital-based data. Informed consent was taken from all participants for all procedures, and the tenets of the Declaration of Helsinki were followed. Apart from baseline characteristics visual acuity at presentation, retinal findings on dilated fundus evaluation and DSA imaging results were collected and tabulated according to the subgroups of CCFs.

### Definitions

CCFs was classified according to Barrow *et al*. based on DSA^2^.

Type A (high flow) CCFs defined as, when there was a direct communication between the internal carotid artery cavernous segment and the cavernous sinus.

Type B (low flow) CCFs defined as, when there is an abnormal communication between the cavernous sinus and meningeal branches of the internal carotid artery.

Type C (low flow) CCFs defined as, when there was an abnormal communication between the cavernous sinus and meningeal branches of the external carotid artery.

Type D (low flow) CCFs defined as, when there was an abnormal communication between the cavernous sinus, and one or more meningeal branches of the internal carotid artery and external carotid artery.

VI was classified based on the WHO criterion^9^. No to mild VI was defined as visual acuity less or equal to 6/18. Moderate VI was defined as visual acuity less than 6/18 but better than or equal to 6/60. Severe VI was defined as visual acuity less than 6/60 but better than or equal to 3/60. Blindness was defined as visual acuity less than 3/60.

### Statistical analysis

Statistical analysis was performed using statistical software (SPSS for Windows, version 21.0 SPSS Science, Chicago, IL). Tests for normality were performed; as the data were not normally distributed, non-parametric tests were used. We compared the proportions of various retinal manifestation and diseases and VI between the two groups using the Chi-Square test. We compared the mean age of patients between the two groups using the Mann-Whitney test. A p value of less than 0.05 was set as statistical significance.

## Results

Of the 48 patients, 8 (16.7%) patients had a high flow CCFs, and 40 (83.3%) patients had low flow CCFs. Table 1 shows the baseline characteristics of high flow (type A) and low flow groups (type B, C, and D). The mean age of the patients was 26.6 +/− 10.51 years (mean +/− SD) and 50.3 +/− 17.1 years (mean +/− SD) in the high and low flow group respectively (p < 0.05). There were 6 (75%) and 22 (55%) males in the high flow and low flow group, respectively. History of trauma was more common in high flow as compared to low flow group, 87.5% Vs 5% (p < 0.05). In high flow, 1 of 8 cases (12.5% prevalence) were bilateral Vs in low flow, 3 of 37 cases (8.1% prevalence) were bilateral (p > 0.05).

**Table 1:** Baseline Characteristics of Study Subjects.

Characteristics	High flow	Low flow			P value (high vs. low)
	A*(*n* = 8)	B*(*n* = *6)*	C*(*n* = 7)	D*(*n* = 27)	
Mean age (years)	26.6	54.0	42	55.3	**<0.0001**
Gender (M/F)^†^	6/2	4/2	4/3	14/13	0.440
History of trauma	7 (87.5%)	0	1 (14.3%)	1 (3.7%)	**<0.0001**
Laterality (Unilateral)	7 (87.5%)	6 (100%)	7 (100%)	24 (88.9%)	1.000

A, B, C, D: Classification by Barroww’s based on DSA; ^†^M: Male, F: Female.

Table 2 shows the retinal morphological features in CCFs. Retinal vein dilatation was found in 5 (63.5%) patients and 21 (52.5%) patients in the high flow and low flow groups, respectively, (p < 0.710). In the high flow group, 3 (37.5%) patients had intra-retinal hemorrhage, and 1 (12.5%) patients had pre-retinal hemorrhage, 1 (12.5%) patient had vitreous hemorrhage (secondary to Terson syndrome). There was 1 (12.5%) patient with disc pallor (secondary to ischemic optic neuropathy) and 1 (12.5%) patient with disc hyperemia (patients presented with central retinal artery occlusion [CRAO]).

**Table 2:** Retinal Morphological Features in High Vs Low Flow CCF.

Characteristics	High flow	Low flow			P value (high vs. low)
	A*(n = *8)*	B*(n = *6)*	C* (n = *7)*	D*(n = *27)*	
Retinal vein dilatation	5 (62.5%)	4 (66.7%)	4 (57.1%)	13 (48.1%)	0.710
Intraretinal Hemmorhages	3 (37.5%)	3 (50%)	1 (14.3%)	8 (29%)	1.000
Preretinal Hemmorhage	1 (12.5%)	1 (16.8%)	0	2 (7.4%)	0.189
Vitreous Hemmorhage	1 (12.5%)	0		0	0.167
Macular edema	0	1 (16.8%)	0	3 (11.1%)	0.594
Choroidal detachment	0	0	0	1 (3.7%)	1.000
Retinal detachment	0	0	0	1 (3.7%)	1.000
Disc Hyperaemia	1 (12.5%)	3 (50%)	1 (14.3%)	5(18.5%)	1.000
Disc pallor	1 (12.5%)	0	0	1 (3.7%)	0.671

*A, B, C, D: Classification by Barroww’s based on DSA.

In the low flow group, 12 (30%) patients had intra-retinal hemorrhage, 3 (7.5%) patients had pre-retinal hemorrhage, 4 (10%) patients had macular edema (two of them were secondary to central retinal vein occlusion (CRVO) and rest had macular edema as an isolated finding), and 1 (2.5%) patient had combined choroidal and retinal detachment. Disc hyperemia was present in 9 (22.5%) patients, and disc pallor was present in 1 (secondary to ischemic optic neuropathy) patient.

Table 3 describes the posterior segment manifestations in CCFs. In the high flow group, there was 1 (12.5%) patient with glaucomatous damage and 1 (12.5%) patient with traumatic CRAO. In the low flow group, there were 2 patients (5%) with CRVO and six patients (15%) with glaucomatous disc damage.

**Table 3:** Posterior segment manifestations in high Vs low flow CCF.

Disease	High flow	Low flow			P value (high vs. low)
	A* (n = *8)*	B* (n = *6)*	C*(n = *7)*	D* (n = *27)*	
CRVO^†^	0	0	0	2 (7.4%)	1.000
Glaucomatous cupping	1 (12.5%)	2 (33.3%)	1 (14.3%)	3 (11.1%)	1.000
CRAO^‡^	1 (12.5%)	0	0	0	0.167
ION	1 (12.5%)	0	0	1 (3.7%)	
TON	2 (25.0%)	0	0	0	

^*^A, B, C, D: Classification by Barroww’s based on DSA, ^†^CRVO: Central retinal vein occlusion,

^‡^CRAO: Central retinal artery occlusion.

Table 4 shows the levels of VI in high flow and low flow groups. Overall, 6 (75.0%) and 10 (25.0%) patients had visual impairment in high and low flow groups, respectively. In the high flow group, 2 (25%) patients had no to mild VI, 1 (12.5%) patient had a moderate VI, 1 (12.5%) patient had severe VI and the remaining 4 (50%) patients were blind. In the low group, 30 (75%) patients had a no to mild visual impairment, 5 (12.5%) had a moderate visual impairment, 2 (5%) had a severe visual impairment and remaining 3 (7.5%) were blind. There was a statistically significant difference in the proportions of VI between the two groups (p = 0.017).

**Table 4:** Distribution of Levels of Visual Impairment in CCF.

Visual Impairment^†^	High flow	Low flow			p value (high Vs low)
	A*(n = *8)*	B*(n = *6)*	C* (n = *7)*	D*(n = *27)*	
Mild	2 (25%)	4 (66.7%)	5 (71.4%)	21 (77.7%)	*0*.*017*
Moderate	1 (12.5%) *ION* ^††^	2 (33.3%) *Stasis retinopathy*	1 (14.3%) *Stasis retinopathy*	2 (7.4%) *Stasis retinopath*y	
Severe	1 (12.5%) *GON* ^‡‡^	0	1 (14.3%) *GON* ^‡‡^	1 (3.7%) *ION* ^††^	
Blindness	4 (50%) [*2-TON*^^^*;1-TS*^$^ 1- CRAO^**^]	0	0	3 (11.1%) *[2-CRVO*^‡^*; 1- RD* + *CD*^#^]	

^*^A, B, C, D: Classification by Barroww’s based on DSA, ^†^No to mild visual impairment: visual acuity less that or equal to 6/18. Moderate visual impairment: visual acuity less than 6/18 but better than or equal to 6/60.Severe visual impairment: visual acuity less than 6/60 but better than or equal to 3/60. Blindness: visual acuity less than 3/60.

^‡^CRVO: Central retinal vein occlusion; ^**^CRAO: Central retinal artery occlusion; ^††^ION.

ischemic optic neuropathy; ^‡‡^GON: Glaucomatous optic neuropathy; ^#^RD: Retinal detachment + CD: Choroidal detachment; ^^^TON: Traumatic optic neuropathy; ^$^TS: Terson Syndrome.

The causes of VI in the high flow group were: traumatic optic neuropathy (33%), vitreous hemorrhage (16.7%), CRAO (16.7%), glaucomatous optic neuropathy (16.7%) and ischemic optic neuropathy (16.7%).

The causes of VI in the low flow group were: stasis retinopathy (50%), CRVO (20%), glaucomatous optic neuropathy (10%), ischemic optic neuropathy and combined choroidal and retinal detachment (10%).

## Discussion

We report the posterior segment manifestations in 48 consecutive cases of CCFs which were proven and classified on DSA. Of the total 48 patients, the high flow and low flow group constituted 16.7%and 83.3%, respectively. Recently, Tan *et al*. found that the high flow group constituted 18% of the total 45 angiograms proven CCFs patients cohort^10^. Although most earlier studies report that 70–90% of CCFs are of the direct type, this study did not find the same^11,12^. This difference may reflects the lower incidence of head injuries due to improved traffic regulations and greater sensitivity of modern imaging in the diagnosis of low flow dural CCFs types that remained previously undiagnosed. We found that majority of the low flow CCFs were of type D (67.5%) followed by type C (17.5%) and type B (15%). Preechawat *et al*. in their study found a similar result: type D (71%), type C (15%), and type B (14%)^6^. Since the low flow CCF patients (83.3%)) have mild signs and symptoms, a majority of them are diagnosed late, leading to VI in many. We found 10 (25.0%) patients in low flow group had a visual impairment. Therefore, a higher index of suspicion and lower threshold for imaging might be appropriate.

We found young males and trauma (5 had a history of road traffic accident and remaining 2 had a history of assault with an iron rod) to be significant risk factors for high flow CCFs. Our results are in agreement with the previous studies^12,13^. Preechawat *et al*. found no gender predisposition and higher mean age in the low flow CCFs patients, as found in our study^6^.

We had one patient who was referred for left-sided peri-orbital swelling of two-week duration following a road traffic accident. On examination, the left eye had no perception of light secondary to a CRAO of recent onset^14^. Another patient presented with a sudden onset proptosis, redness and gross diminution of vision associated with severe head injury. Ophthalmic evaluation and ultrasound B-scan showed the presence of vitreous hemorrhage. Imaging showed the sub-dural hematoma and diffused cerebral edema following a road traffic accident (Terson syndrome). Serious ocular damage may be present in patients with head injury. These patients can present with various uncommon combination of manifestations, and early recognition and appropriate treatment can salvage vision. A multi-disciplinary team approach is necessary for patients with head trauma. We had one patient with non-traumatic high flow CCF. Non-traumatic type A CCFs can be caused by rupture of a preexisting aneurysm of the cavernous segment of the internal carotid artery, can be iatrogenic (after craniofacial diagnostic and therapeutic procedures)^15–19^, or can be spontaneous with a predisposing vasculopathy, such as systemic hypertension or a connective tissue disorder (namely Ehler-Danlos syndrome or fibromuscular dysplasia)^15–17^. Interestingly, our patient was a young hypertensive pregnant lady who developed an acute onset of unilateral proptosis and redness associated with diplopia during the third trimester. She was evaluated, and imaging showed type A CCF. She underwent successful embolization following which her symptoms resolved. There are similar case reports in the literature of CCFs in pregnancy^18,19^.

The varied clinical presentation of CCFs depends on the anatomy, hemodynamics and size of the CCF fistula. The retinal manifestations are the secondary to venous stasis and impaired retinal flow leading to ischemia. Therefore, the retinal manifestation ranges from mild stasis retinopathy to frank CRVO^1,20,21^. The most common retinal finding in our study was dilatation of the retinal veins which was present in 5 (62.5%) patients and 21 (52.5%) patients in the high flow and the low flow group, respectively. Our results are comparatively lower than that reported previously in the literature by Henderson and Schneider (76.5%)^22^. A possible reason is this may have been missed, given that it is a relatively subtle finding.

Since most of the patients have unilateral presentation (91.7%) and are of low flow CCFs (87.5%), the high prevalence of the retinal vein dilatation in CCF patients has important implications. Patients with low flow CCFs have mild signs and symptoms and may be misdiagnosed and poorly responsive to attempted therapy. Additionally they may manifest with a variety of presentations including chronic conjunctivitis, blepharoconjunctivitis, ocular hypertension, secondary glaucoma, episcleritis, thyroid eye disease and others. We recommend a careful dilated fundus examination and comparison with the other eye, which might provide an important clue to the ophthalmologist to clinch the correct diagnosis.

Retinal hemorrhages in CCFs patients are usually flame-shaped or punctate, but in rare cases, sub-hyaloid (pre-retinal) or vitreous hemorrhage have also been reported^1,11,23,24^. This can be with or without associated CRVO and its complications. We found 15 (31.2%) and 5 (10.4%) patients had intra-retinal and pre-retinal hemorrhage, respectively. CRVO has been well reported with CCF patients and represents the most severe manifestation of stasis retinopathy^25,26^. Visual impairment in CRVO is due to ischemic maculopathy, macular edema and epiretinal membrane. When proliferative retinopathy sets in, it can result in vitreous hemorrhage and tractional retinal detachment involving the macula contributing to vision loss. We had two patients in the low flow group who developed CRVO. Our observation is in agreement with previous reports of CRVO in low flow CCF^27,28^. Although the venous stasis is more in the high flow CCF, early recognition and treatment might explain the lower incidence noted in this group, unlike the low flow group. Moreover, CRVO has been reported in CCF patients after embolization^29^. Interestingly we had two (4.2%) patients who developed macular edema without an associated CRVO. Since both the patients had vein dilatation and few retinal haemorrhages, this macular edema could be secondary to an impending CRVO, where macular edema has been reported^30^.

We had one patient with type D CCF who had combined choroidal and retinal detachment. He developed sudden onset proptosis, redness and gross diminution of vision following trabeculectomy surgery and was treated elsewhere as supra-choroidal haemorrhage^31^. Likewise, Nagaki *et al*. had reported a case of CCF developing following cataract surgery^32^. The choroid undergoes significant remodeling in patients with CCF as have been described by Sutoh *et al*.^33^, In the past, choroidal detachment and retinal detachment have been described in CCF patients^34,35^. Choroidal detachment in CCFs is thought to be the result of severe choroidal vascular congestion, stasis and increased fluid transudation in these patients secondary to raised venous pressure. In some patients, choroidal thickening and detachment can be associated with secondary angle closure glaucoma^36^.

We had 10 (20.8%) patients who had disc hyperemia. Stasis retinopathy can result in disc hyperemia to frank disc edema and has been described in CCF patients in the literature^1,7,37^. Saatci *et al*. reported one case of optic disc neovascularization with minimal peripheral retinal ischemia in a case of traumatic high fIntravitreal injection of ranibizumab was successful in treating this condition^38^.

We found glaucomatous cupping in 9 (14%) patients. Glaucomatous optic neuropathy was seen in 2 (3.8%) patients. The need for intra ocular pressure monitoring and anti-glaucoma therapy cannot be overemphasized^6,23,39^. We had 2 (3.8%) patients who had optic disc pallor with a history of delayed visual loss over a period of a few weeks. In patients with long-standing fistulas, the optic nerve gets damaged from compression by a distended cavernous sinus or from retrobulbar ischemia, causing ischemic optic neuropathy^1,40^.

VI in high flow CCFs can be either immediate or delayed. Of the 6 (75%) patients with VI in the high-flow, immediate and delayed visual loss was seen in 4 (50%) and 2 (25%) patients, respectively. Immediate causes were: traumatic optic neuropathy (25%), vitreous haemorrhage (12.5%), and central retinal artery occlusion (12.5%). Delayed causes were ischemic optic neuropathy (12.5%) and glaucomatous optic damage (12.5%).

VI in low flow CCFs is of delayed onset secondary to chronic hypoxia-induced retinal dysfunctions. Chronic hypoxia results from: (a) alterations in hemodynamics caused by the fistula results in a decrease in effective ophthalmic artery perfusion pressure (b) arterialization of the orbital venous system results in an increase in venous pressure, and (c) thrombosis of the ophthalmic venous system. VI was seen in 10 (25.0%) patients in low flow group and resulted from: stasis retinopathy (12.5%), central retinal vein occlusion (5.0%), ischemic optic neuropathy (2.5%), glaucomatous optic damage (2.5%), and combined retinal and choroidal detachment (2.5%).

**Figure 1:**
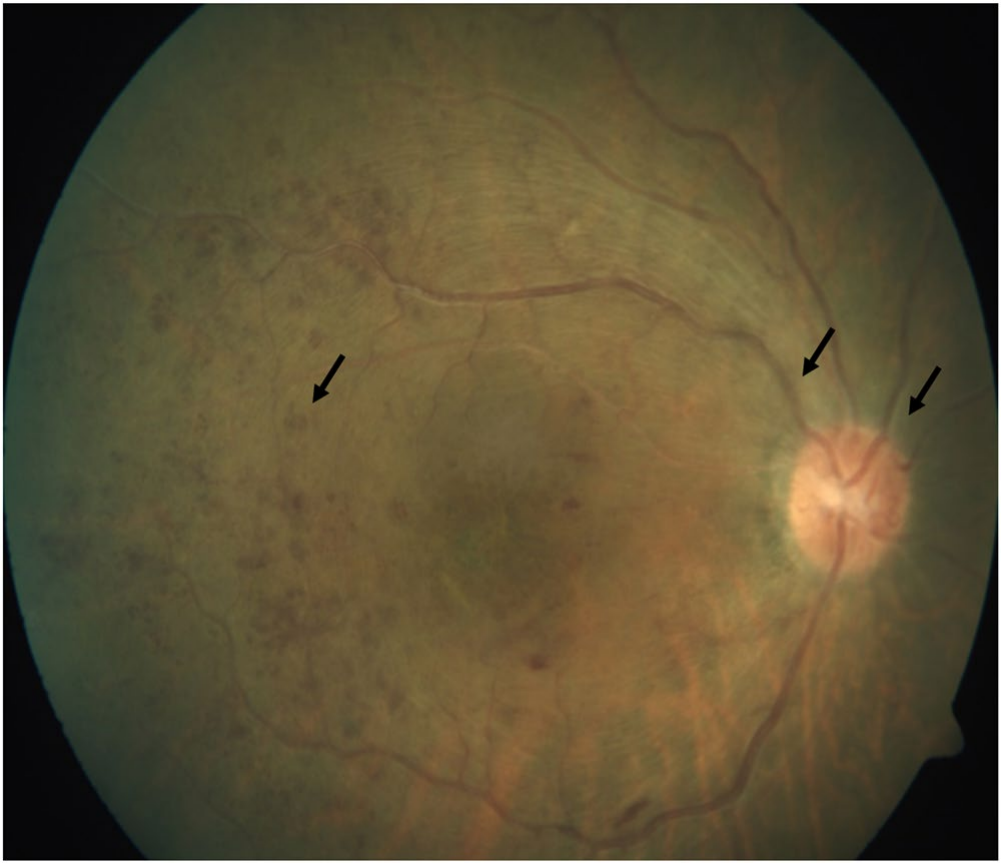
Fundus photograph of the right eye of a low-flow CCF patient showing disc hyperemia, retinal vein dilatation and intra-retinal hemorrhage (3 point sign).

In the low flow group, we had five patients with moderate VI resulting from stasis retinopathy with features of: retinal vein dilatation, intra-retinal hemorrhages, and disc hyperemia (Fig. 1). These cases are secondary to varied posterior segment changes, which the ophthalmologist needs to carefully asses for and arrange treatment accordingly. Retinal manifestations of these five patients were compared with 30 patients with no VI. Interestingly, none of the 30 patients had these three features together. This finding has important implications. Although retinal manifestation in stasis retinopathy ranges from retinal vein dilatation on the one hand to CRVO on the other, VI seems to start midway between the two extremes, “3 point sign”. While none of the findings in isolation were predictive of visual loss, when present together they were associated with VI. Unlike in high flow CCFs, there is always a dilemma when to intervene in low flow CCFs patients. The “3 point sign” could help clinicians decide when intervention should be seriously considered. Frequent dilated fundus examinations are important to recognize the “3 point sign” at the earliest opportunity. The potential importance of the “3 point sign” for prognosis and treatment of CCF needs to be validated in future studies.

The strength of this study lies in the DSA proven cases included in this study and in-depth analysis of varied posterior segment changes, and the incidence of visual impairment and its causes. The identification of “3 point sign” is a novel finding of this study, not described before.

The basic retrospective design is a limitation of the study. We did not find any significant difference in the posterior segment findings in high flow and low flow group. We attribute it to: (a) small number of patients in the high flow group, (b) low flow CCF patients being misdiagnosed frequently, a dilated retinal evaluation was done in a relatively advanced stage of the disease. Future research is required to support this finding of ours. Also, as ours is a single tertiary care specialty ophthalmic Centre, the patients after diagnosis of CCF were referred to the neurosurgical unit and were lost to follow-up at our hospital. Thus, we were unable to analyze the results of the treatment in our cohort.

In conclusion, the incidence of VI in CCF is high, and most of these are secondary to varied posterior segment changes described earlier, which needs to be carefully looked for and treated accordingly. A common yet crucial finding of retinal vein dilatation can help to clinch the diagnosis in some patients, with similar manifestation. Although the anterior segment changes are of most concern to the patients, frequent dilated fundus evaluation is important. “3 sign point” must be identified at the earliest to prevent visual impairment. Future research is necessary to validate this important finding in similar large case series.

## Data Availability

The datasets generated during and/or analysed during the current study are available from the corresponding author on reasonable request.
